# Serum Cytokine Profiles Associated with Inflammation and Tumor Progression in Crohn’s Disease and Colorectal Cancer

**DOI:** 10.3390/ijms27052156

**Published:** 2026-02-25

**Authors:** Michał Święch, Justyna Lorenc-Góra, Małgorzata Muc-Wierzgoń, Elżbieta Świętochowska, Paweł Kowalczyk, Zenona P. Czuba, Dariusz Waniczek

**Affiliations:** 1 Clinical Department of General and Colorectal Surgery and Multiple Trauma, St Barbara Regional Specialist Hospital No 5, Medical University of Silesia in Katowice, 40-055 Katowice, Poland; swiech_michal@op.pl; 2 Department of Imaging Diagnostics, St Barbara Regional Specialist Hospital No 5, 41-214 Sosnowiec, Poland; lorencgora.j@gmail.com; 3 Department of Internal Diseases, Propedeutics and Emergency Medicine, Faculty of Public Health in Bytom, Medical University of Silesia in Katowice, Piekarska 18, 44-902 Bytom, Poland; mwierzgon@sum.edu.pl; 4 Department of Medical and Molecular Biology, School of Medicine with the Division of Dentistry, Medical University of Silesia in Katowice, 40-055 Katowice, Poland; eswietochowska@sum.edu.pl; 5 Department of Animal Nutrition, The Kielanowski Institute of Animal Physiology and Nutrition, Polish Academy of Sciences, Instytucka 3, 05-110 Jabłonna, Poland; 6 Department of Microbiology and Immunology, Faculty of Medical Sciences in Zabrze, Medical University of Silesia in Katowice, 40-055 Katowice, Poland; zczuba@sum.edu.pl; 7 Department of Oncological Surgery, Faculty of Medical Sciences in Zabrze, Medical University of Silesia in Katowice, 40-055 Katowice, Poland

**Keywords:** Crohn’s disease, colorectal cancer, cytokines, IL-9, MIP-1β, PDGF-β, chronic inflammation, tumorigenesis, principal component analysis

## Abstract

Chronic inflammation is a recognized driver of colorectal cancer (CRC) development, particularly in patients with Crohn’s disease (CD). This study aimed to explore serum inflammatory cytokine profiles in patients with Crohn’s disease and colorectal cancer and to assess their associations with disease activity and tumor-related features. Seventy-eight participants were included: 24 with CD, 31 with CRC, and 23 healthy controls. Serum levels of 27 cytokines were measured using the Bio-Plex Pro Human Cytokine 27-plex Assay. Principal component analysis identified three cytokine clusters (factors). Factor 2, comprising IL-9, MIP-1β, and PDGF-β, was significantly elevated in CD patients compared to controls (*p* < 0.001), showed intermediate levels in CRC patients, and positively correlated with fecal calprotectin (R = 0.44; *p* = 0.04), indicating an association with local intestinal inflammation. In CRC patients, Factor 1, comprising key Th1/Th17 cytokines (IL-1β, IL-2, IL-4, IL-5, IL-15, IL-17A, FGF-B, GM-CSF, IFN-γ, TNF-α, and VEGF), reflecting pro-inflammatory and cell-mediated immune signaling, correlated with lymph node metastasis (τ = 0.27; *p* = 0.03), while Factor 2 showed a trend toward a negative correlation with tumor histological grade (τ = −0.22; *p* = 0.09). Factor 3, encompassing regulatory and hematopoietic cytokines, did not differ significantly between groups (*p* = 0.21). These findings suggest that IL-9, MIP-1β, and PDGF-β reflect inflammatory activity and may be involved in inflammation-associated tumorigenic processes in the gut. Serum profiling of selected cytokines may provide biologically relevant information and support further investigation of inflammation-associated immune patterns in CD and CRC. Further studies are warranted to validate their clinical utility and to elucidate their mechanistic role in inflammation-driven colorectal carcinogenesis.

## 1. Introduction

Colorectal cancer (CRC) is one of the most common malignancies worldwide. It ranks as the third most frequently diagnosed cancer in both sexes and the second leading cause of cancer-related mortality [1,2]. Well-established risk factors for CRC include diet, physical inactivity, tobacco smoking, alcohol consumption, advanced age, and genetic predisposition [3,4]. Chronic inflammation has also been shown to increase the likelihood of CRC development [5,6]. In patients with inflammatory bowel disease (IBD), including Crohn’s disease (CD) and ulcerative colitis (UC), disease severity has been linked to the balance between pro-inflammatory and anti-inflammatory cytokines [7,8]. Studies investigating the association between pro-inflammatory cytokines and carcinogenesis have demonstrated that patients with Crohn’s disease (CD) are at an increased risk of developing colorectal cancer (CRC). Inflammatory mediators such as TNF-α, IL-6, and IL-1β further contribute to a pro-tumorigenic microenvironment by enhancing angiogenesis and immune cell infiltration [9,10]. Experimental models of colitis-associated cancer have provided direct evidence that persistent inflammation precedes and promotes tumor initiation and progression, supporting the concept that chronic inflammatory activity itself is a key driver of tumorigenesis in the colon [11,12,13]. Together, these findings suggest that long-standing inflammatory signaling in CD creates conditions conducive to colorectal tumor development.

Recent years have seen growing interest in inflammatory cytokines as potential prognostic biomarkers in CRC. Circulating cytokines may reflect the activity of the tumor inflammatory microenvironment, clinical disease stage, and potential response to therapy [14,15].

Various pro-inflammatory cytokines have been analyzed regarding their role in CD pathogenesis and disease activity. Previous studies have demonstrated strong correlations between platelet-derived growth factor (PDGF-β) and interleukin-6 (IL-6) with clinical features of CD. such as endoscopic disease activity and erythrocyte sedimentation rate (ESR) [16,17]. Associations have also been observed between IL-9, IL-4, IL-5, and IFN-γ with IBD progression and their potential utility in assessing disease stage.

Calprotectin is a well-established biomarker of intestinal inflammation and one of the most extensively studied diagnostic parameters in IBD. It shows higher sensitivity in CD and correlates with disease activity; its fecal concentration increases during CD flare-ups, paralleling elevated CRP and ESR. It also helps differentiate inflammatory from non-inflammatory bowel conditions [18,19,20,21,22,23].

Comparing cytokine profiles in CD and CRC may allow the identification of cytokines with diagnostic potential. It may also enable the recognition of an inflammatory CRC phenotype characterized by a distinct clinical course and potentially different responses to treatment, including positive responses to biologic therapy similar to those observed in CD. Despite extensive research on cytokines in both CD and CRC, comparative analyses of serum cytokine profiles across these conditions remain limited, particularly with respect to identifying shared and distinct inflammation-associated immune patterns that may reflect the transition from chronic inflammation to colorectal carcinogenesis.

The immune response in inflammatory bowel disease and colorectal cancer is mediated by complex, highly interconnected cytokine networks rather than by isolated mediators acting independently. In this context, multivariate approaches such as principal component analysis (PCA) provide a valuable exploratory tool for reducing data dimensionality and identifying dominant patterns of cytokine co-variation that may reflect coordinated immune responses [9,12,16].

PCA enables the identification of cytokine clusters representing shared variance across the dataset, thereby highlighting immune signatures that may correspond to distinct inflammatory, regulatory, or tissue-remodeling states without presupposing predefined biological pathways [12,16]. Such cytokine patterns have been increasingly applied in immunological and oncological research to characterize systemic inflammation, immune surveillance, and tumor-associated immune remodeling [21,22,23,24,25,26,27,28,29,30,31,32]. Importantly, these statistically derived components do not represent direct mechanistic signaling pathways but rather reflect higher-order organization of immune mediators influenced by disease activity, host-related factors, and the tumor microenvironment [9,21].

Therefore, the application of PCA in the present study was intended as a hypothesis-generating approach to explore systemic cytokine signatures potentially linking inflammatory activity with neoplastic transformation, while acknowledging the exploratory nature and biological complexity of such analyses.

The aim of this study was to explore serum inflammatory cytokine profiles in patients with Crohn’s disease and colorectal cancer and to investigate their associations with disease activity and tumor-related features.

## 2. Results

### 2.1. Characteristics of the Study and Control Groups

#### 2.1.1. Crohn’s Disease Group

The CDG consisted of 24 patients, aged 22 to 78 years (mean 45; SD ± 15.48), including 14 women (58%) and 10 men (42%). These patients were hospitalized due to CD exacerbation or surgical complications of the disease (intestinal fistulas or gastrointestinal obstruction). Fecal calprotectin levels were assessed in all patients using a commercial enzyme-linked immunosorbent assay (ELISA), and the inflammatory activity of CD was evaluated using the Crohn’s Disease Activity Index (CDAI). Clinical parameters of this group are presented in (Table 1).

In the analyzed Crohn’s disease group (CDG), the mean Crohn’s Disease Activity Index (CDAI) score was 226, indicating moderate-to-severe disease activity. The median CDAI of 214 and the upper quartile of 274 suggest that the majority of patients exhibited markedly active inflammation. These high values are consistent with the criteria for hospitalization, where CDAI scores > 220 indicate disease exacerbation and may warrant inpatient treatment [24,25]. Clinical disease activity assessed by CDAI was also reflected in fecal calprotectin levels: 83.3% of patients exceeded the reference range, and the mean calprotectin concentration was more than nine times higher than the upper limit (483.02 µg/g vs. ≤50 µg/g).

#### 2.1.2. Colorectal Cancer Group

The CRCG consisted of 31 patients, aged 46 to 85 years (mean 65; SD ± 14.56). This group included 18 men (58.06%) and 13 women (41.94%); see (Table 2).

According to the [33], the most frequently observed tumor stage in the analyzed CRC group was T3 (64.52%), with regional lymph node metastases most commonly at stage N2 (61.29%). Distant metastases (M1) were present in 41.94% of patients. The majority of tumors exhibited a moderate histologic grade (G2—68.97%). The most common clinical stage was IV (38.71%), followed by stage III (32.26%); see (Table 3).

#### 2.1.3. Control Group

The CG consisted of 23 patients, aged 50 to 85 years (mean 65; SD ± 9), including 17 women (73.91%) and 6 men (26.09%). These were patients admitted for elective surgery for lower limb varicose veins or inguinal hernia repair, in whom IBD, malignancy, autoimmune diseases, and decompensated chronic diseases were excluded; see (Table 4).

In the colorectal cancer group (CRCG), men predominated (58.06%), whereas in the Crohn’s disease group (CDG), women accounted for 58% of participants, and in the control group (CG), women represented 73.91%. Differences in sex distribution between the analyzed groups approached statistical significance (*p* = 0.06); see (Table 5).

Values of the analyzed clinical and laboratory parameters differed significantly between the study groups and the control group; see (Table 6). The median age was lowest in the Crohn’s disease group (CDG—43.5 years) and highest in the colorectal cancer group (CRCG—68.0 years); *p* < 0.0001. Significant differences were also observed in body mass index (BMI), with the lowest in the CDG (21.85 kg/m^2^) and the highest in the CRCG (26.45 kg/m^2^), *p* < 0.0001. Hematocrit (HT) levels were highest in the control group (41.8%) and differed significantly between groups (*p* < 0.001). In contrast, white blood cell count (WBC) did not show significant differences between groups (*p* = 0.96).

The mean age of patients in the CDG was significantly lower than in the other groups, reflecting the natural course of Crohn’s disease, which typically manifests earlier than CRC. This discrepancy may influence the interpretation of certain inflammatory and hematologic parameters, such as hematocrit, which decreases with age. In addition, BMI in older adults should be interpreted cautiously, as moderately elevated values may reflect age-related changes in body composition rather than excess adiposity.

Obesity is increasingly common among individuals with CD. However, higher BMI is generally associated with lower disease activity, indicating disease stabilization [26,27,28]. In the CDG, BMI was lower compared to the other groups, which may be related to the specific selection of patients for this study—they were individuals with chronic disease complicated by, among others, intestinal fistulas and subobstruction, as well as patients in an active disease flare [29,30].

In contrast, the CRCG predominantly comprised patients with advanced disease (over 70% at stages III and IV according to the TNM classification), mostly with histologic grades G2 or G3, indicating biologically significant tumor progression. This may influence cytokine profiles and the intensity of the inflammatory response. The presence of distant metastases (M1) in 42% of patients also suggests systemic inflammatory changes that may modulate the levels of measured biomarkers.

The control group was selected based on the absence of inflammatory, neoplastic, and autoimmune diseases, increasing the reliability of comparisons. However, the high proportion of women in this group may affect hematocrit and leukocyte composition, although these parameters show only a moderate dependence on sex.

After adjustment for age, sex, and BMI in the multivariate linear regression model, Factor 2 (IL-9, MIP-1β, PDGF-β) remained significantly associated with the disease group. Compared with controls, patients with Crohn’s disease exhibited significantly higher Factor 2 values (β = 0.62 and *p* = 0.001), while colorectal cancer patients showed intermediate but still significant levels (β = 0.34 and *p* = 0.021). None of the demographic or anthropometric variables (age, sex, and BMI) were independently associated with Factor 2, confirming that the observed differences were not driven by confounding demographic factors but rather reflected disease-related immune activation.

Table 7 presents a multivariate linear regression analysis evaluating the independent association between clinical group and Factor 2 (IL-9, MIP-1β, PDGF-β) after adjustment for age, sex, and BMI. CD and CRC groups were compared to healthy controls. Statistically significant associations are highlighted in bold.

Comparison of component values between the study groups is presented in Table 8. Factor 2 showed significantly higher values in the CDG compared to CRCG and CG (*p* < 0.001, Kruskal–Wallis test). Its level was also significantly higher in the CRCG than in the CG, although lower than in the CDG.

For Factor 1, borderline significant differences were observed between the groups (*p* = 0.07). with the highest levels noted in CD patients. The lowest median Factor 1 values were observed in the control group. Factor 3 values did not differ significantly between groups (*p* = 0.21) (Table 8).

Additionally, in the CDG, correlations were assessed between fecal calprotectin levels and the Crohn’s Disease Activity Index (CDAI) with the values of the three components; see Table 9 and Table 10.

Using Kendall’s Tau correlation coefficient, the relationships between component values and tumor stage according to the TNM classification, as well as histologic grading (G), were analyzed in CRC patients. The results are presented in Table 11. Factor 1 showed a significant positive correlation with the N feature (regional lymph node involvement; τ = 0.27; *p* = 0.03). and a borderline significant correlation with the T feature (tumor size/invasion; τ = 0.24; *p* = 0.06). Factor 2 exhibited a borderline significant negative correlation with tumor grade (G; τ = −0.22; *p* = 0.09). All other correlations were not statistically significant.

Table S3 (in Supplementary Materials) summarizes the LLOD for each cytokine and the proportion of samples below the detection limit in each study group. Overall, low or undetectable cytokine concentrations were most frequent in the healthy control group (CG), consistent with expected physiological baseline levels. These low values were included in principal component analysis (PCA) after imputation as half of the LLOD [7,8,16], ensuring comprehensive analysis of the data set [USW4].

## 3. Discussion

Crohn’s disease (CD) and colorectal cancer (CRC) are clinically distinct entities but share overlapping pathogenic mechanisms related to chronic immune activation and dysregulated inflammatory signaling. Persistent cytokine-driven inflammation in CD has been implicated in epithelial damage, impaired mucosal healing, and increased risk of inflammation-associated colorectal carcinogenesis. In this context, the present study applied principal component analysis (PCA) as an exploratory, multivariate approach to identify coordinated patterns of systemic cytokine variation across CD, CRC, and healthy control groups.

Among the three extracted cytokine components, Factor 2—comprising IL-9, MIP-1β (CCL4), and PDGF-β—emerged as the most discriminative immune signature. Factor 2 values were highest in patients with CD, intermediate in CRC patients, and lowest in healthy controls, suggesting a graded relationship between inflammatory burden and disease state. Importantly, Factor 2 showed a significant positive correlation with fecal calprotectin levels in CD patients, supporting its association with active intestinal inflammation. This finding indicates that systemic expression of IL-9, MIP-1β, and PDGF-β reflects inflammatory activity linked to mucosal immune processes rather than isolated circulating changes.

IL-9 is a cytokine primarily produced by Th9 cells and plays a complex role in mucosal immunity. In CD, elevated IL-9 levels have been associated with disease activity and impaired epithelial barrier integrity. Experimental and clinical studies suggest that IL-9 may exert context-dependent effects in colorectal carcinogenesis, potentially promoting epithelial proliferation and survival through activation of JAK/STAT and PI3K/AKT signaling pathways, while also exhibiting anti-tumor properties in certain tumor microenvironments. The intermediate IL-9-related signal observed in CRC patients in the present study may reflect a transitional immune state between chronic inflammation and established neoplasia.

PDGF-β, another key component of Factor 2, is involved in tissue repair, stromal remodeling, and angiogenesis. In CD, increased PDGF-β expression has been linked to active inflammation and fibrotic changes. In CRC, PDGF-β contributes to tumor growth, angiogenesis, and metastatic potential through activation of PDGFR-mediated signaling cascades, including PI3K/AKT and mTOR pathways. Elevated PDGF-β levels in both CD and CRC groups in this study support the concept that growth factor–driven remodeling processes may bridge chronic inflammation and tumor progression.

MIP-1β (CCL4) is a chemokine produced mainly by activated macrophages and plays a role in leukocyte recruitment to sites of inflammation. Although its role in colorectal carcinogenesis is less well defined, MIP-1β participates in immune cell trafficking and activation of inflammatory signaling pathways such as JAK/STAT and MAPK/ERK. Its inclusion in Factor 2 alongside IL-9 and PDGF-β suggests that coordinated chemotactic and remodeling signals accompany inflammatory activity in both CD and CRC.

In contrast to Factor 2, Factor 1—dominated by Th1/Th17-related cytokines including IL-1β, IFN-γ, IL-17A, TNF-α, and GM-CSF—did not significantly differ between groups but showed meaningful associations within the CRC cohort. Specifically, higher Factor 1 values correlated with regional lymph node involvement, indicating that intensified systemic pro-inflammatory immune activation may be linked to tumor invasiveness and metastatic spread. This observation aligns with previous reports demonstrating that pro-inflammatory cytokines contribute to tumor progression by promoting angiogenesis, extracellular matrix remodeling, and immune evasion.

Factor 3, encompassing regulatory and hematopoietic cytokines such as IL-7, IL-10, IL-13, and G-CSF, did not differ significantly between study groups and showed no clear associations with disease activity or tumor-related parameters. This suggests that systemic regulatory immune signals are relatively preserved across inflammatory and neoplastic conditions or that their effects are more context-specific and not captured by circulating measurements alone.

Taken together, the present findings support the concept that chronic intestinal inflammation and colorectal tumor progression share partially overlapping systemic immune signatures. The identification of a cytokine cluster centered on IL-9, MIP-1β, and PDGF-β highlights a coordinated inflammatory–remodeling profile that is most pronounced in CD and partially retained in CRC. While these results provide insight into immune patterns potentially linking inflammation and carcinogenesis, they should be interpreted within the exploratory framework of the study. Further longitudinal and mechanistic investigations are required to clarify the temporal dynamics and functional significance of these cytokine signatures in inflammation-associated colorectal cancer development.

### Limitations

This study has several important limitations that should be acknowledged when interpreting the results. First, the cross-sectional design and relatively small sample size preclude causal inference and limit the generalizability of the findings. The identified cytokine patterns should therefore be regarded as exploratory and hypothesis-generating rather than predictive or clinically actionable.

Second, the study groups differed significantly in age, sex distribution, and body mass index, which may independently influence systemic cytokine profiles. Although multivariable analyses adjusting for age, sex, and BMI confirmed that Factor 2 (IL-9, MIP-1β, and PDGF-β) remained independently associated with disease group, residual confounding related to immunosenescence, sex-dependent hormonal status, and metabolic factors cannot be fully excluded.

Third, pharmacological treatment represents a potential confounding factor. Patients with Crohn’s disease were heterogeneous with respect to prior or ongoing exposure to corticosteroids, immunosuppressive agents, and biologic therapies, all of which are known to modulate circulating cytokine levels. Although subgroup analyses did not reveal statistically significant differences in PCA-derived cytokine factors between treated and untreated patients, detailed stratification by treatment type, duration, and dosing was not feasible in this cohort. Therefore, treatment effects cannot be fully disentangled from disease-related immune signatures.

Fourth, cytokine concentrations below the lower limit of detection occurred more frequently in the control group. Although values below LLOD were handled using standard imputation procedures (half-LLOD) to allow inclusion in multivariate and PCA analyses, this approach may introduce some degree of uncertainty, particularly for cytokines with low baseline circulating levels.

Finally, principal component analysis identifies patterns of statistical co-variation rather than mechanistic signaling pathways. Although biologically plausible labels were assigned to facilitate interpretation, the extracted factors reflect complex immune network interactions influenced by systemic inflammation, disease activity, and host-related factors. Only cytokines with strong factor loadings (≥0.70) were considered primary contributors, while cytokines with moderate loadings were not over-interpreted.

Taken together, these limitations underscore the exploratory nature of the present study. Future investigations should include larger, age- and sex-matched cohorts, longitudinal designs, treatment-stratified analyses, and complementary mechanistic approaches to validate the clinical and biological relevance of the identified cytokine signatures.

Finally, the scope of the analysis was limited to the cytokines included in the multiplex panel, and other immune mediators relevant to intestinal inflammation and tumor biology were not assessed.

## 4. Materials and Methods

### 4.1. Materials

A total of 78 patients were enrolled in the study. They were divided into three groups: patients diagnosed with Crohn’s disease (CDG), patients with colorectal cancer (CRCG), and healthy control subjects (CG).

Inclusion criteria for patients with CD and CRC were: age over 18 years, any disease stage, and consent to participate in the study (Ethics Committee SUM approval no. KNW/0022/KB1/21/I/10). Inclusion criteria for patients with Crohn’s disease and colorectal cancer were: age ≥ 18 years, diagnosis of CD or CRC, regardless of disease stage, and written informed consent to participate in the study. The study was approved by the Ethics Committee of the Medical University of Silesia (approval no. KNW/0022/KB1/21/I/10).

Exclusion criteria for patients with CD and CRC included: severe systemic or metabolic diseases (except obesity as an isolated disorder), previous radiotherapy or chemotherapy, other malignancies, chronic active inflammatory diseases (current or history), including diagnosed IBD in CRC patients, and reoperations due to the primary disease.

### 4.2. Methods

Based on the literature, a panel of 27 cytokines with potential prognostic significance or associations with CRC development was selected [7,9,16,31]: IL-1β, IL-1RA, IL-2, IL-4, IL-5, IL-6, IL-7, IL-8, IL-9, IL-10, IL-12 (p70), IL-13, IL-15, IL-17α, Eotaxin, FGF-β, G-CSF, GM-CSF, IFN-γ, IP-10, MCP-1 (MCAF), MIP-1α, MIP-1β, PDGF-β, RANTES, TNF-α, and VEGF. Based on the literature, we selected a broad cytokine panel to capture key biological axes implicated in CRC, including systemic inflammation and immunoregulation (e.g., IL-1β, IL-6, TNF-α, and IL-10/IL-1RA), T helper cell-related immune signaling (IL-2, IL-4, IL-5, IL-12 (p70), IL-13, IL-15, and IL-17α), chemokine-mediated immune cell recruitment (IL-8, MCP-1, MIP-1α/β, RANTES, IP-10, and Eotaxin), and growth/angiogenic and hematopoietic mediators (VEGF, PDGF-β, FGF-β, G-CSF, and GM-CSF). The selected cytokines participate in key immunological pathways involving Th1, Th2, Th9, Th17, and Treg responses. They primarily include chemokines and growth factors associated with intestinal inflammation, epithelial regeneration, and tumor progression. Consequently, the relatively large number of analytes reflects an a priori, pathway-oriented profiling strategy rather than exploratory “single-marker” testing.

Venous blood samples were collected from all patients (CDG, CRCG, and CG) in the morning after an overnight fast, using clot tubes. Following centrifugation, the obtained serum was transferred into sterile tubes and stored at −70 °C until laboratory analysis.

All samples were analyzed in duplicate, and the final concentration was calculated as the mean of the two measurements. If the standard deviation (SD) of a sample exceeded the accepted range, the measurement was repeated.

#### 4.2.1. Determination of Selected Cytokine Concentrations in Serum

Serum cytokine concentrations were determined using a multiplex bead-based immunoassay technique, performed according to the manufacturer’s instructions (Bio-Rad Laboratories, Inc. Hercules, CA, USA). The method is based on antibody-coated microspheres with distinct spectral signatures, enabling the simultaneous detection of multiple cytokines within a single sample.

Briefly, serum samples were incubated with cytokine-specific antibody-coated beads, followed by incubation with biotinylated detection antibodies and fluorescent streptavidin–phycoerythrin. After washing steps to remove unbound reagents, bead-bound complexes were analyzed using the Bio-Plex 200 system (Bio-Rad Laboratories, Inc. Hercules, CA, USA). Data acquisition and concentration calculations were performed using also by Bio-Plex 200 system software (Bio-Rad Laboratories, Inc. Hercules, CA, USA), in accordance with the applied standards.

##### Cytokine Measurement

Serum concentrations of cytokines were measured using a commercially available multiplex bead-based immunoassay (Bio-Plex Pro Human Cytokine 27-plex Assay, Bio-Rad Laboratories, Hercules, CA, USA; cat. no. M500KCAF0Y), according to the manufacturer’s instructions. The panel simultaneously quantified 27 cytokines and chemokines, including pro-inflammatory, anti-inflammatory, and adaptive immunity–related mediators.

This multiplex approach was selected to enable comprehensive profiling of systemic inflammatory cytokine patterns while minimizing sample volume and inter-assay variability. All samples were analyzed in duplicate, and fluorescence was measured using the Bio-Plex 200 system. Data acquisition and concentration calculations were performed with Bio-Plex 200 system software.

The 27-plex panel was selected to enable broad, exploratory profiling of cytokines involved in adaptive immunity, inflammation, chemotaxis, and tissue remodeling, which are relevant to both Crohn’s disease and colorectal cancer; see Supplementary Table S1.

##### Calprotectin Measurement

Fecal calprotectin levels were measured in all patients with Crohn’s disease using a commercially available ELISA kit, according to the manufacturer’s instructions. Calprotectin values were incorporated into the statistical analysis and specifically correlated with PCA-derived cytokine factors to explore associations between systemic cytokine signatures and local intestinal inflammation [19,20,21,22,23,24,25,26,27,28,29,30,31,32,34,35,36,37,38,39,40,41,42,43,44,45,46,47,48,49,50,51,52,53,54].

##### Multivariable Analysis Adjusting for Demographic and Anthropometric Confounders

To assess the independence of the observed effects, an additional multivariable analysis was performed using linear regression (ANCOVA). Factor 2 (IL-9, MIP-1β, PDGF- β,) was used as the dependent variable, while age, sex, and BMI were included as covariates. The disease group was entered as an independent factor; see Supplementary Table S2. Factor 2 was derived from standardized values of IL-9, MIP-1β, and PDGF- β. Age, sex, and BMI were included as covariates in the model (see Supplementary Materials, Figures S1–S11).

The overall model was statistically significant (F-test for the full model, *p* < 0.05) and explained a meaningful proportion of variance in Factor 2 (R^2^ and adjusted R^2^ indicating good model fit). Diagnostic analyses confirmed the absence of multicollinearity and verified that model assumptions were met.

##### Handling of Values Below the Lower Limit of Detection (LLOD)

Values below the lower limit of detection (LLOD) for each cytokine, as specified by the Bio-Plex Pro Human Cytokine 27-plex Assay manufacturer, were handled according to recommended procedures. Specifically, undetectable values were imputed as half of the LLOD for statistical analyses [7,8,16]. This approach minimizes bias and allows inclusion of all samples in multivariate and PCA analyses, consistent with standard practice in multiplex cytokine profiling [12,16].

#### 4.2.2. Statistical Analysis

Data are presented as mean ± standard deviation for normally distributed variables and as median with interquartile range for non-normally distributed variables. Normality was assessed using the Shapiro–Wilk test. For comparisons of quantitative variables. Either the Mann–Whitney U test or the parametric Student’s *t*-test was used, depending on the distribution. Correlations between variables were evaluated using either Pearson’s or Spearman’s rank correlation coefficient, depending on the distribution of the data. Factor analysis of the measured cytokines was performed using the principal component method. Three factors were extracted and normalized; Varimax rotation was applied to maximize the explained variance. Factor scores were further analyzed in the context of patient groups and their clinical and pathological parameters.

**Figure 1:**
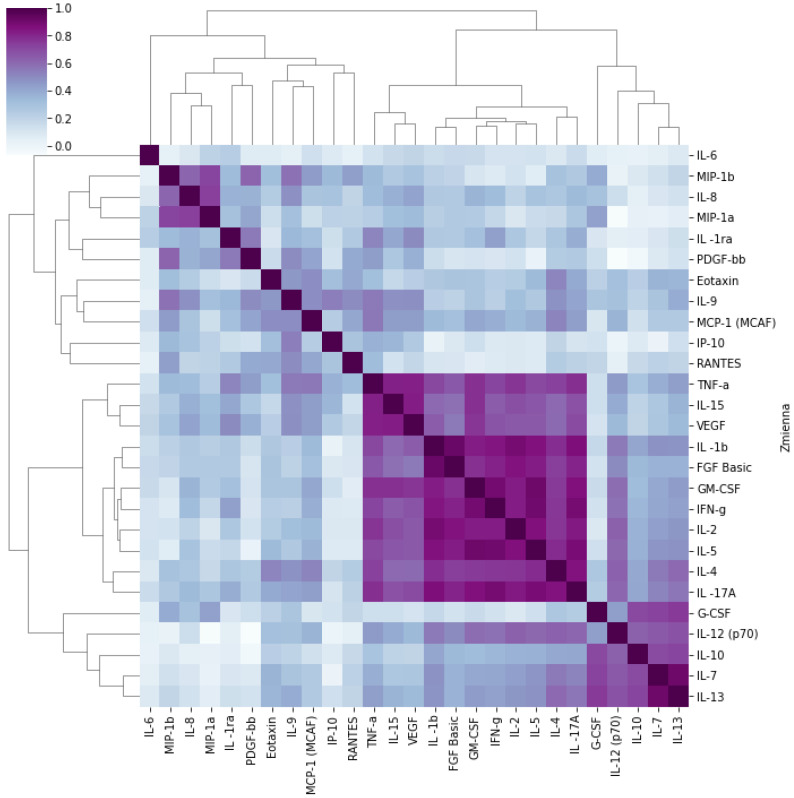
Graphical representation of correlations between the analyzed cytokines. Hierarchically clustered (Pearson correlation, n = 78).

Statistical analyses were performed using STATISTICA 13 software (Tibco Software Inc. Palo Alto, CA, USA). A *p*-value < 0.05 was considered statistically significant (Figure 1).

The exact number of patients in each group

Crohn’s disease (CD): n = 24;

Colorectal cancer (CRC): n = 31;

Control group: n = 23.

This approach was used to assess correlations between individual cytokines. Due to the large number of results obtained, principal component factor analysis was performed. This allowed the identification of three factors, i.e., groups of positively correlated cytokines, which together explained 67.17% of the system’s variance; see (Table 12).

Using a factor loading threshold of ≥0.70, three components (i.e., groups of cytokines) were identified, indicating a strong association of each variable with the corresponding latent factor.

The components were as follows:

1. **Factor 1:** IL-1β, IL-2, IL-4, IL-5, IL-15, IL-17α, FGF-β, GM-CSF, IFN-γ, TNF-α, VEGF;
2. **Factor 2:** IL-9, MIP-1β, PDGF-β;
3. **Factor 3:** IL-7, IL-10, IL-13, G-CSF.

Factor 1 includes pro-inflammatory and effector cytokines, typical of Th1- and Th17-type responses. The highest factor loadings in this group were observed for IFN-γ (0.93), GM-CSF (0.92), IL-5 (0.92), IL-2 (0.91), IL-1β (0.89), IL-17A (0.88), as well as angiogenesis- and proliferation-related factors such as FGF-β, TNF-α, and VEGF. These cytokines indicate activation of inflammatory and adaptive immune pathways, particularly involving T lymphocytes.

Factor 2 primarily represents chemokines and cytokines involved in leukocyte migration and tissue remodeling. This group includes, among others, MIP-1β (0.85), PDGF-β (0.74), IL-9 (0.73), IL-8 (0.68), and MIP-1α (0.69). This pattern may reflect local activation of monocytes, granulocytes, and platelets, as well as recruitment of immune cells to sites of inflammation.

Factor 3 encompasses regulatory and hematopoietic cytokines, such as IL-13 (0.87), IL-7 (0.87), IL-10 (0.83), and G-CSF (0.85). These cytokines are involved in immune cell differentiation and maturation, as well as balancing the inflammatory response, suggesting an immunomodulatory role for this group.

The three extracted components (Factor 1, Factor 2, and Factor 3) were subsequently compared between the study groups (CDG, CRCG, and CG) (Figure 2; see Materials and Methods, Figures S1–S11, Table S2).

Prior to factor extraction, the suitability of the dataset for principal component analysis (PCA) was assessed using the Kaiser–Meyer–Olkin (KMO) measure of sampling adequacy and Bartlett’s test of sphericity. The KMO value was 0.81, indicating very good sampling adequacy, while Bartlett’s test was statistically significant (*p* < 0.001), confirming that the correlation matrix was appropriate for factor analysis.

Principal component analysis was performed using Varimax rotation to enhance factor interpretability. Factors were extracted based on the following criteria:

1. Eigenvalues > 1.0 (Kaiser criterion);
2. Inspection of the scree plot;
3. Cumulative variance explained.

Factor loadings ≥ 0.70 were considered strong and used for factor interpretation. Loadings between 0.50 and 0.69 were regarded as moderate and interpreted cautiously. The selected three-factor solution explained 67.17% of the total variance, providing an optimal balance between model simplicity and explanatory power.

**Figure 2:**
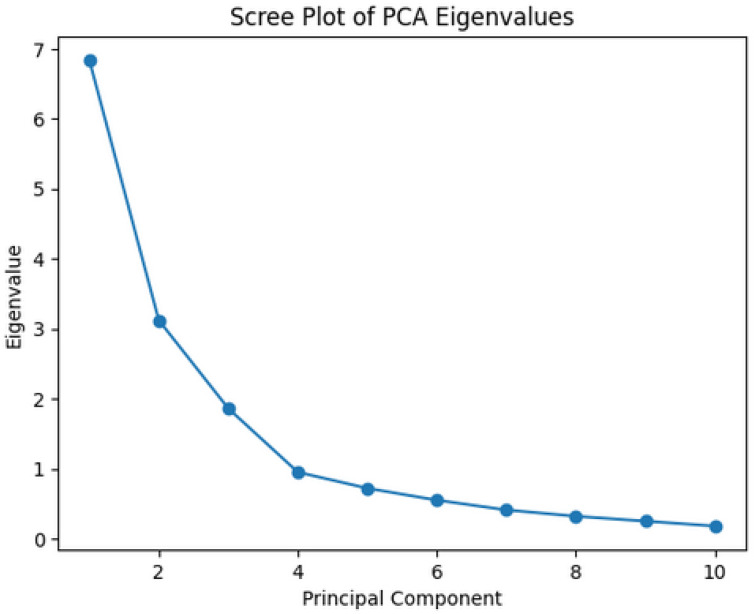
Scree plot of eigenvalues obtained from principal component analysis. The inflection point after the third component supports the retention of a three-factor solution.

The Kaiser–Meyer–Olkin measure of sampling adequacy was 0.81, and Bartlett’s test of sphericity was highly significant (*p* < 0.001), confirming the suitability of the dataset for principal component analysis. Based on eigenvalues greater than 1.0 and inspection of the scree plot (Figure 2), three principal components were extracted, which together explained 67.17% of the total variance. The eigenvalues for Factors 1, 2, and 3 were 6.84, 3.12, and 1.86, respectively. The scree plot demonstrated a clear inflection point after the third component, supporting the retention of a three-factor solution.

Subgroup comparisons between biologically treated and untreated Crohn’s disease patients were performed using the Mann–Whitney U test or Student’s *t*-test, as appropriate. A *p*-value < 0.05 was considered statistically significant.

## 5. Conclusions

In this exploratory study, multivariate analysis of serum cytokine profiles identified a distinct immune signature characterized by IL-9, MIP-1β, and PDGF-β that differentiated patients with Crohn’s disease and colorectal cancer from healthy controls. The association of this cytokine cluster with fecal calprotectin in Crohn’s disease and with selected tumor-related features in colorectal cancer suggests a potential link between systemic inflammatory activity and inflammation-associated processes during intestinal disease progression. However, due to the cross-sectional design and methodological limitations, these findings should be interpreted with caution and considered hypothesis-generating rather than clinically actionable. Further longitudinal and mechanistic studies are warranted to validate the biological relevance and potential translational value of the identified cytokine patterns.

## Figures and Tables

**Table 1:** Clinical parameters of the CDG.

Characteristic	Min	Max	Median	Q1	Q3	Mean	SD
Age, years	22	78	43.5	31	58.5	45	15.8
BMI, kg/m^2^	13.5	32	21.8	19.5	24.1	22.1	4.27
WBC, tys/µL	3.65	25.1	7.04	4.96	9.66	8.06	5.02
Ht, %	25.7	47.4	36	30.5	40.5	35.6	6.12
CDAI	88	408	214	165	274	226	92.5
Calprotectin, µg/g	1.57	1161.71	317.76	145.85	880.31	483.02	403.61

BMI = body mass index; WBC = white blood cells; Ht = hematocrite; and CDAI = Crohn’s Disease Activity Index.

**Table 2:** Clinical parameters of the CRCG.

Parameters	Min	Max	Median	Q1	Q3	Mean	SD
Age, years	46	85	68.00	59.00	73.00	65	14.56
BMI, kg/m^2^	18	45.4	26.45	24.45	29.50	27.36	5.28
WBC, tys/µL	2.57	11.53	6.56	5.09	8.37	6.76	2.07
Ht, %	28	42.8	37.95	36.15	40.10	38.37	3.22

BMI = body mass index; WBC = white blood cells; and Ht = hematocrite.

**Table 3:** Clinicopathological parameters of the CRCG.

T		
	n	%
1	1	3.23
2	7	22.58
3	20	64.52
4	3	9.68
N	n	%
0	9	29.03
1	3	9.68
2	19	61.29
M	n	%
0	18	58.06
1	13	41.94
G	n	%
1	1	3.45
2	20	68.97
3	8	27.59
Stage	n	%
I	5	16.13
II	4	12.90
III	10	32.26
IV	12	38.71

n = number of patients; T = tumor size/invasion; N = regional lymph node involvement; M = distant metastases; G = histologic grade; and Stage = clinical stage.

**Table 4:** Clinical parameters of CG.

Parameters	Min	Max	Median	Q1	Q3	Mean	SD
Age, years	50	85	64.00	59.50	71.50	65.4	9.98
BMI, kg/m^2^	20.1	44.1	24.30	23.40	27.60	26	4.85
WBC, tys/µL	3.56	10.8	7.32	5.42	8.30	6.94	1.84
Ht, %	29.9	45.8	41.80	40.10	43.70	41.4	3.48

BMI = body mass index; WBC = white blood cells; Ht = hematocrite.

**Table 5:** Sex distribution in patients with Crohn’s Disease (CDG), colorectal cancer (CRCG), and the control group (CG).

	CG		CRCG		CDG		*p*-Value
	n	%	n	%	n	%	
Female	17	73.91	13	41.94	14	58%	0.06
Male	6	26.09	18	58.06	10	42%	

n = number of patients.

**Table 6:** Clinical and laboratory characteristics of patients with Crohn’s Disease (CDG), colorectal cancer (CRCG), and the control group (CG).

Parameters	CDG (N = 24)	CRCG (N = 31)	CG (N = 23)	*p*-Value
Age, years	43.50	68.00	64.00	0.0001
BMI, kg/m^2^	21.85	26.45	24.30	0.0001
WBC, tys/µL	7.04	6.56	7.32	0.96
HT, %	36.05	37.95	41.80	0.00

Data are presented as the median value. BMI = body mass index; WBC = white blood cells; and HT = hematocrite.

**Table 7:** Multivariate analysis of Factor 2 (IL-9, MIP-1β, PDGF-β) with adjustment for demographic and anthropometric variables.

Variable	β Coefficient	95% CI	Standard Error	*p*-Value
Group (CD vs. CG)	0.62	0.28–0.96	0.17	0.001
Group (CRC vs. CG)	0.34	0.05–0.63	0.15	0.021
Age (years)	−0.09	−0.21–0.03	0.06	0.143
Sex (male vs. female)	0.11	−0.18–0.40	0.15	0.452
BMI (kg/m^2^)	0.07	−0.04–0.19	0.06	0.201

Model statistics: R^2^ = 0.41. Adjusted R^2^ = 0.37, F = 6.92, *p* < 0.001.

**Table 8:** Comparison of component values between study groups (median and interquartile range Q1–Q3) (Kruskal–Wallis test, *p* < 0.05).

	CG			CRCG			CDG			*p*
Variable	Median	Q1	Q3	Median	Q1	Q3	Median	Q1	Q3	
Factor 1	−0.18	−0.22	−0.12	−0.26	−0.32	−0.13	0.03	−0.29	0.3	0.07
Factor 2	−0.99	−1.19	−0.33	−0.1	−0.55	0.33	0.77	−0.07	1.51	0
Factor 3	−0.32	−0.44	−0.08	−0.15	−0.31	0.04	−0.26	−0.47	0.21	0.21

**Table 9:** Correlation between component values and fecal calprotectin levels in the CDG (n = 24) (Spearman’s Correlation Coefficient, *p* < 0.05).

Variable	Spearmen’s Rank Correlation	
	R	*p*
Factor 1 & Calprotectin	−0.04	0.87
Factor 2 & Calprotectin	0.44	0.04
Factor 3 & Calprotectin	−0.04	0.88

**Table 10:** Correlation between components and the CDAI in the CDG (Spearman’s Correlation Coefficient, *p* < 0.05).

Variable	Spearman’s Rank Correlation	
	R	*p*
Factor 1 & CDAI	0.06	0.79
Factor 2 & CDAI	0.39	0.07
Factor 3 & CDAI	0.24	0.28

CDAI = Crohn’s Disease Activity Index.

**Table 11:** Correlations between component values and CRC stage.

Variable	Tau Kendall’s Correlation	
	Tau	*p*
Factor 1 & T	0.24	0.06
Factor 1 & N	0.27	0.03
Factor 1 & G	0.06	0.66
Factor 2 & T	−0.08	0.53
Factor 2 & N	−0.03	0.8
Factor 2 & G	−0.22	0.09
Factor 3 & T	0.02	0.87
Factor 3 & N	−0.11	0.37
Factor 3 & G	0.12	0.38

T = tumor size; N = lymph node involvement; and G = histological tumor grade.

**Table 12:** Factor loadings of the analyzed cytokines.

Variable	Factor Loadings (Normalized Varimax Rotation); Extracted: Principal Components (Highlighted Loadings > 0.70)		
	Factor 1	Factor 2	Factor 3
IL-1b	0.89	0.09	0.25
IL-1RA	0.33	0.53	−0.12
IL-2	0.91	0.05	0.21
IL-4	0.76	0.26	0.39
IL-5	0.92	0.03	0.24
IL-6	0.16	0.16	−0.03
IL-7	0.3	0.05	0.87
IL-8	0.22	0.68	0.02
IL-9	0.2	0.73	0.26
IL-10	0.23	−0.02	0.83
IL-12 (p70)	0.57	−0.1	0.69
IL-13	0.32	0.12	0.87
IL-15	0.74	0.39	0.03
IL-17A	0.88	0.23	0.29
Eotaxin	0.19	0.39	0.37
FGF Basic	0.86	0.08	0.16
G-CSF	−0.07	0.29	0.85
GM-CSF	0.92	0.13	0.16
IFN-γ	0.93	0.08	0.16
IP-10	0.06	0.52	0.05
MCP-1 (MCAF)	0.38	0.55	0.13
MIP-1a	0.09	0.69	0.01
MIP-1b	0	0.85	0.14
PDGF-β	0.08	0.74	−0.06
RANTES	−0.02	0.58	0.22
TNF-a	0.78	0.45	0.12
VEGF	0.75	0.47	0.02

## Data Availability

The original contributions presented in this study are included in the article. Further inquiries can be directed to the corresponding authors.

## Supplementary Materials

The following supporting information can be downloaded at: https://www.mdpi.com/article/10.3390/ijms27052156/s1.

## Abbreviations

The following abbreviations are used in this manuscript:

AKT	Protein kinase B
BMI	Body mass index
CD	Crohn’s disease
CDAI	Crohn’s Disease Activity Index
CDG	Crohn’s disease group
CG	Control group
CRC	Colorectal cancer
CRCG	Colorectal cancer group
CRP	C-reactive protein
UC	Ulcerative colitis
ELISA	Enzyme-linked immunosorbent assay
FGF-β	Fibroblast growth factor beta (basic FGF)
G	Histological tumor grade
G-CSF	Granulocyte colony-stimulating factor
GM-CSF	Granulocyte–macrophage colony-stimulating factor
HT	Hematocrit
IBD	Inflammatory bowel disease
IFN-γ	Interferon gamma
IL	Interleukin
IL-1β	Interleukin 1 beta
IL-1ra	Interleukin 1 receptor antagonist
IL-2	Interleukin 2
IL-4	Interleukin 4
IL-5	Interleukin 5
IL-6	Interleukin 6
IL-7	Interleukin 7
IL-8	Interleukin 8
IL-9	Interleukin 9
IL-10	Interleukin 10
IL-12 (p70)	Interleukin 12 (p70)
IL-13	Interleukin 13
IL-15	Interleukin 15
IL-17A	Interleukin 17A
IP-10	Interferon gamma-induced protein 10
JAK	Janus kinase
MAPK	Mitogen-activated protein kinase
MCAF	Macrophage chemotactic and activating factor
MCP-1	Monocyte chemoattractant protein 1
MIP-1α	Macrophage inflammatory protein 1 alpha
MIP-1β	Macrophage inflammatory protein 1 beta
mTOR	Mammalian target of rapamycin
N	Regional lymph node involvement
OB	Erythrocyte sedimentation rate
PCA	Principal component analysis
PDGF-BB	Platelet-derived growth factor BB
PDGFR-β	Platelet-derived growth factor receptor beta
PI3K	Phosphoinositide 3-kinase
RANTES	Regulated upon activation: normal T cell expressed and secreted
SD	Standard deviation
STAT	Signal transducer and activator of transcription
T	Tumor size/invasion
Th	T helper cell
TNF-α	Tumor necrosis factor alpha
TNM	Tumor–node–metastasis classification
UICC	Union for International Cancer Control
VEGF	Vascular endothelial growth factor
WBC	White blood cell count
