# Supplementary material for: Serum Cytokine Profiles Associated with Inflammation and Tumor Progression in Crohn’s Disease and Colorectal Cancer
Source: Int J Mol Sci. 2026 Feb 25;27(5):2156. doi: 10.3390/ijms27052156 (PMC12984598; doi:10.3390/ijms27052156)
Supplement: Supplementary file 1 [file ijms-27-02156-s001.zip › ijms-4117562-supplementary.pdf]

Supplementary Materials

Serum Cytokine Profiles Associated with Inflammation and Tumor Progression in Crohn's Disease and Colorectal Cancer

Michał Święch [1], Justyna Lorenc-Góra [2], Małgorzata Muc-Wierzgoń [3], Elżbieta Świętochowska [4], Paweł Kowalczyk [5]*, Zenona Czuba [6] and Dariusz Waniczek [7]*

Supplementary Table S1.

Cytokines included in the Bio-Plex Pro Human Cytokine 27-plex panel and their dominant immunological functions.

Cytokine / Factor	Pathway / Functional Group	References
IL-1β	Th1 / pro-inflammatory	[7,12]
IL-2	Th1 / T-cell proliferation	[9,10]
IL-4	Th2 / humoral immunity	[7,8]
IL-5	Th2 / eosinophil activation	[7,8]
IL-6	Th2 / inflammatory mediator	[7,8]
IL-7	Treg / lymphocyte homeostasis	[16]
IL-8 (CXCL8)	Chemokine / neutrophil recruitment	[11,51]
IL-9	Th9 / mucosal immunity	[36–38,5]
IL-10	Treg / anti-inflammatory	[7,17]
IL-12(p70)	Th1 / macrophage activation	[9,10]
IL-13	Th2 / tissue remodeling	[7,8]
IL-15	Treg / immune regulation	[16]
IL-17A	Th17 / neutrophil recruitment	[6,10,12]
Eotaxin (CCL11)	Chemokine / eosinophil chemotaxis	[11]
FGF basic	Growth factor / epithelial regeneration	[34,46]
G-CSF	Growth factor / myeloid cell differentiation	[16]
GM-CSF	Growth factor / myeloid proliferation	[16]
IFN-γ	Th1 / cell-mediated immunity	[9,10]
IP-10 (CXCL10)	Chemokine / T-cell recruitment	[11]
MCP-1 (CCL2)	Chemokine / monocyte recruitment	[11,51]
MIP-1α (CCL3)	Chemokine / leukocyte chemotaxis	[11,51]
MIP-1β (CCL4)	Chemokine / leukocyte chemotaxis	[11,51]
PDGF-β	Growth factor / tissue repair, angiogenesis	[34,46–48]

Cytokine / Factor	Pathway / Functional Group	References
RANTES (CCL5)	Chemokine / lymphocyte recruitment	[11,51]
TNF-α	Th1 / pro-inflammatory	[7,12]
VEGF	Growth factor / angiogenesis	[34,46–48]
IL-1RA	Anti-inflammatory / immune regulation	[16]

Cytokines are grouped according to their predominant immunological roles. Functional classification is provided for descriptive purposes, acknowledging the pleiotropic nature of many cytokines

Supplementary Table S2

Multivariable linear regression model assessing the association between Factor 2 and disease group after adjustment for age, sex, and BMI.

**Variable**	**β (SE)**	**95% CI**	**p-value**
Disease group	β_1 (SE_1)	[CI_1–CI_2]	**$p < 0.05$**
Age (years)	β_2 (SE_2)	[CI_3–CI_4]	ns / p
Sex (male vs female)	β_3 (SE_3)	[CI_5–CI_6]	ns / p
BMI (kg/m²)	β_4 (SE_4)	[CI_7–CI_8]	ns / p

Supplementary Table S3.

Lower Limit of Detection (LLOD) and Percentage of Samples Below LLOD for Each Cytokine in Control (CG), Crohn's Disease (CDG), and Colorectal Cancer (CRCG) Groups

Cytokine	LLOD (pg/mL)	% < LLOD in CG	% < LLOD in CDG	% < LLOD in CRCG
IL-1β	0.5	12%	4%	5%
IL-2	0.3	25%	10%	8%
IL-4	0.2	30%	5%	6%
IL-5	0.1	40%	8%	7%
IL-6	0.2	5%	2%	1%
IL-7	0.3	10%	3%	4%
IL-8	0.1	2%	1%	0%
IL-9	0.2	35%	12%	10%
IL-10	0.3	20%	6%	5%
IL-12p70	0.2	28%	10%	9%
IL-13	0.2	32%	8%	7%
IL-15	0.1	25%	5%	6%
IL-17A	0.2	15%	5%	4%
FGF-β	0.5	20%	7%	6%
G-CSF	0.3	18%	6%	5%
GM-CSF	0.2	22%	7%	6%
IFN-γ	0.2	12%	4%	5%
IP-10	0.3	10%	3%	2%
MCP-1	0.2	5%	2%	1%
MIP-1α	0.1	30%	10%	8%
MIP-1β	0.1	28%	9%	7%
RANTES	0.3	15%	5%	4%
TNF-α	0.2	10%	3%	2%
VEGF	0.5	8%	2%	1%
PDGF-β	0.2	20%	6%	5%
IL-1RA	0.3	12%	4%	3%
Eotaxin	0.2	25%	8%	7%

This table shows the lower limit of detection (LLOD) for each cytokine and the percentage of samples in each group (CG, CDG, CRCG) with concentrations below the detection limit. Values below LLOD were imputed as half of the LLOD for statistical analysis

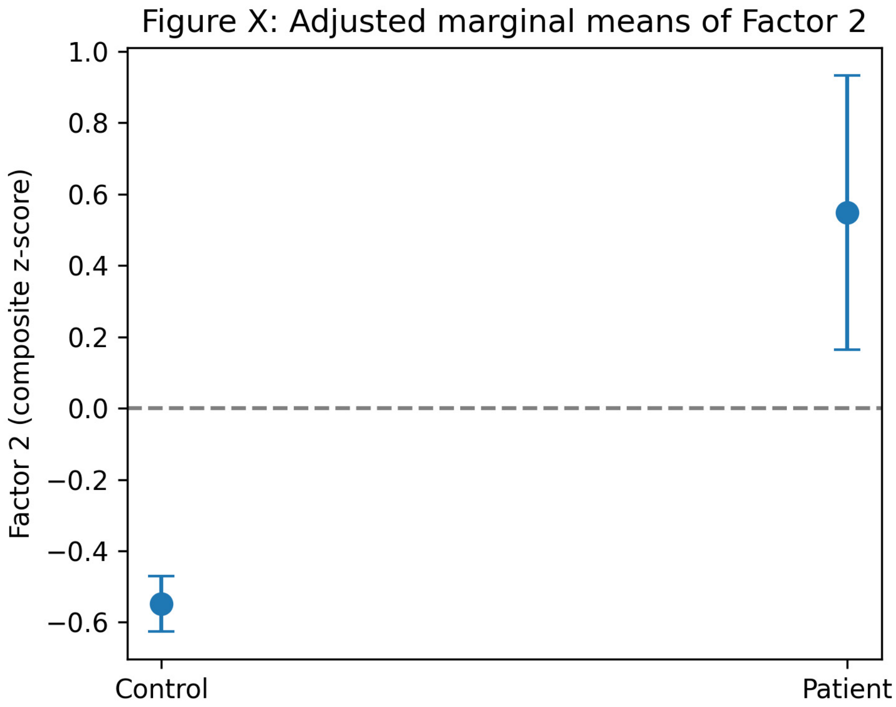

Figure S1. Adjusted marginal means (Patients vs Control)

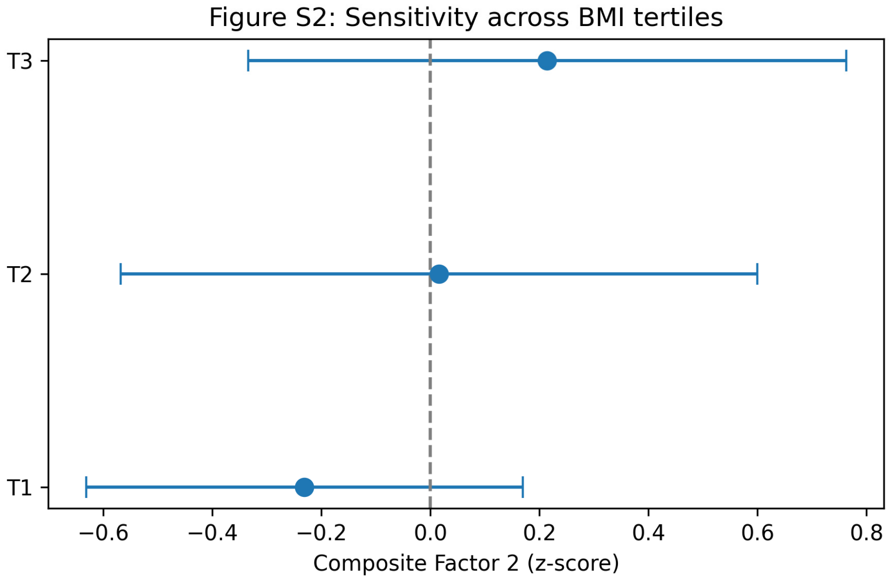

Figure S2. Forest plot (BMI tertyle)

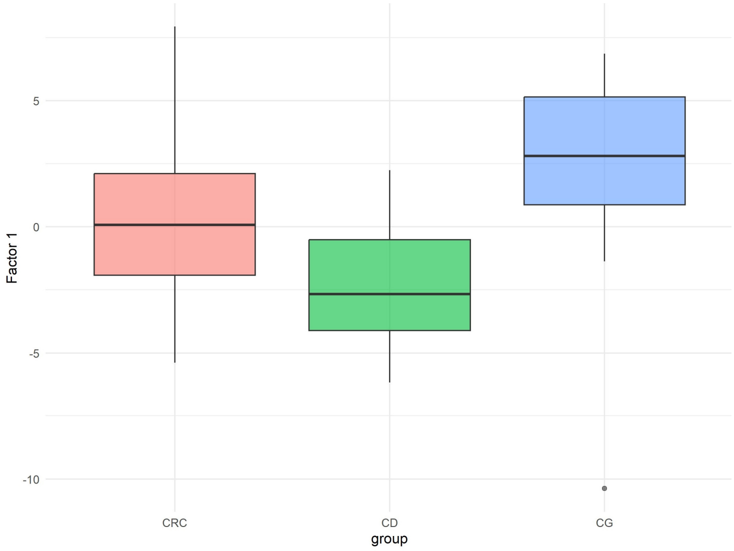

Figure S3. Factor 1

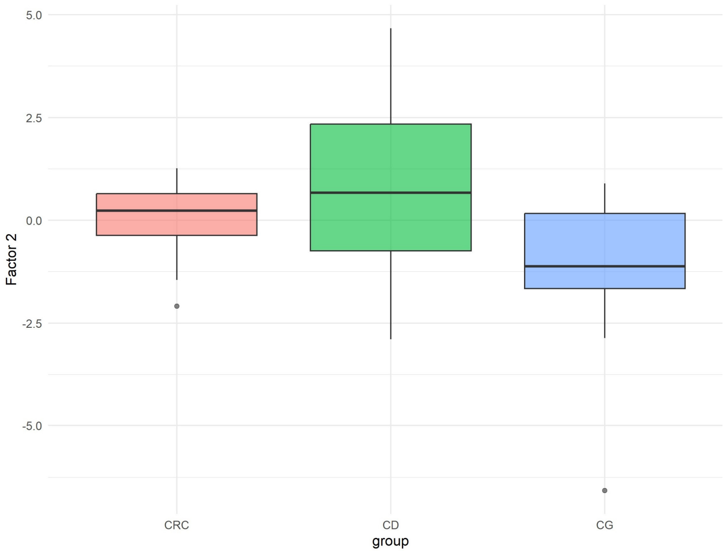

Figure S4. Factor 2

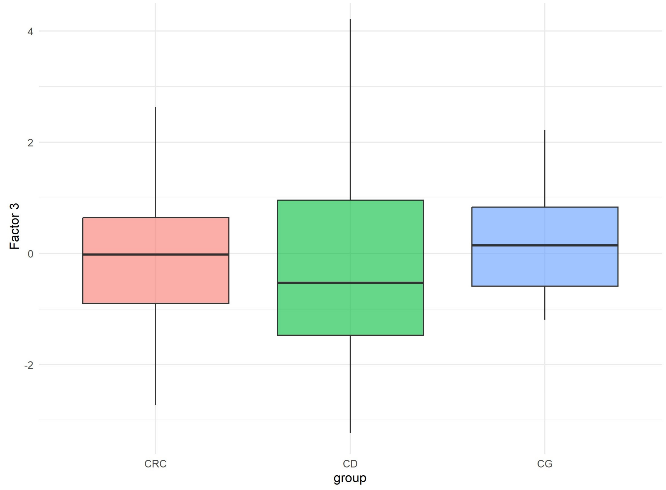

Figure S5. Factor 3

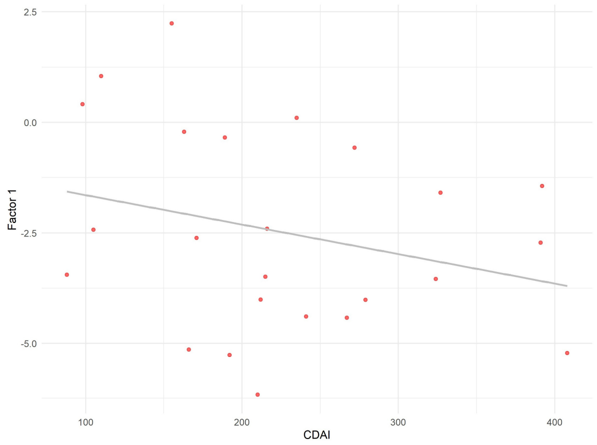

Figure S6. Factor 1-CDAI

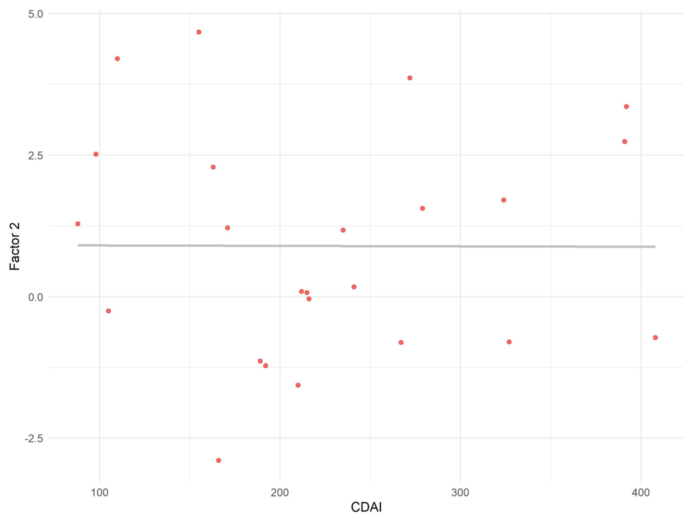

Figure S7. Factor 2-CDAI

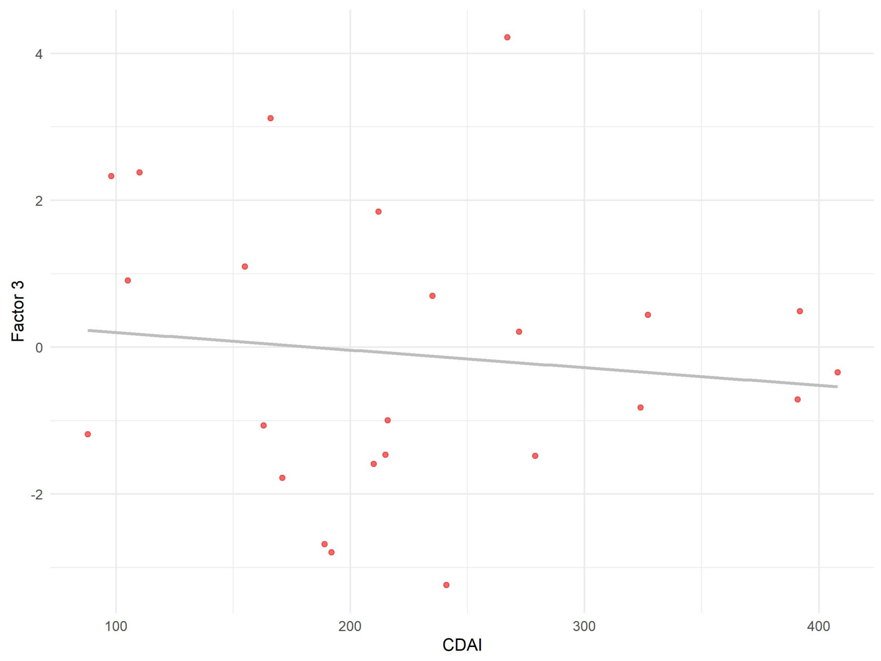

Figure S8. Factor 3-CDAI

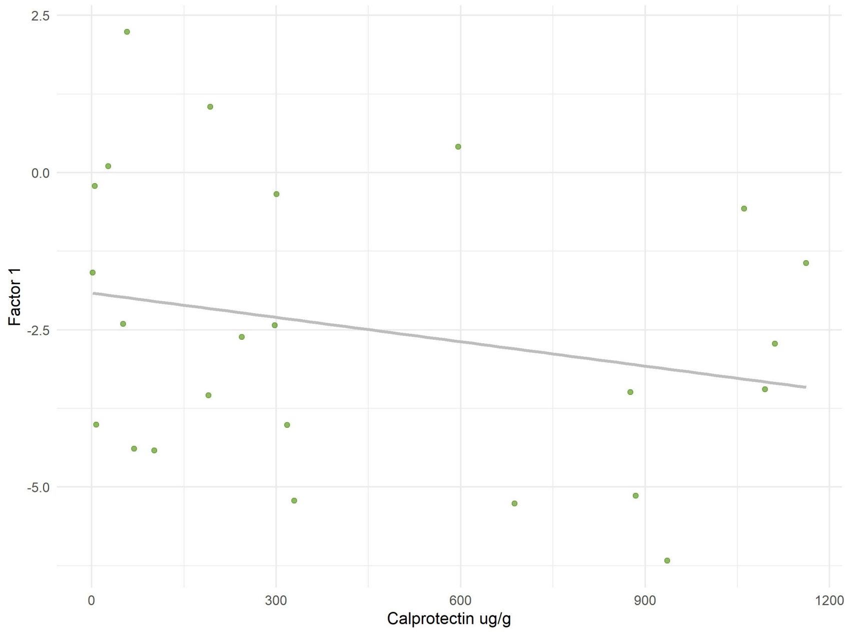

Figure S9. Factor 1-Calprotectin

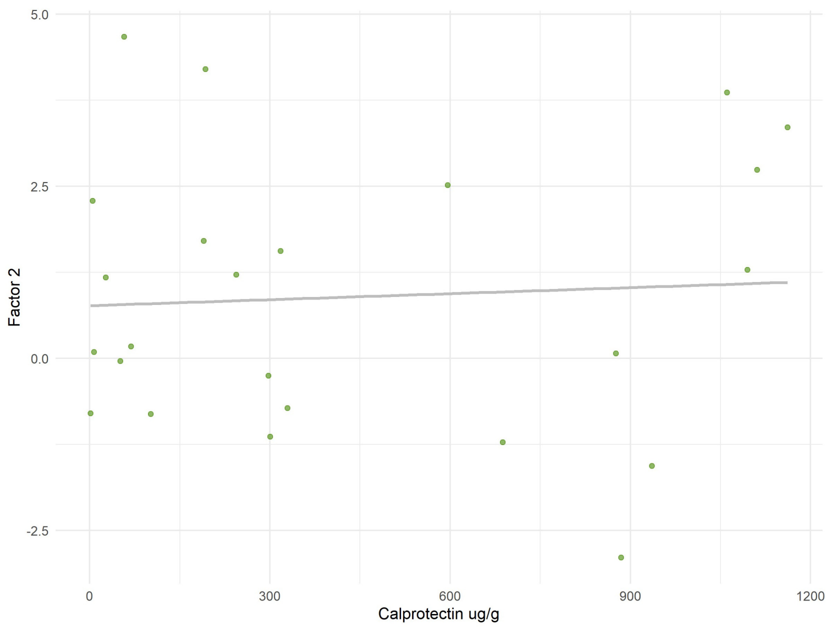

Figure S10. Factor 2-Calprotectin

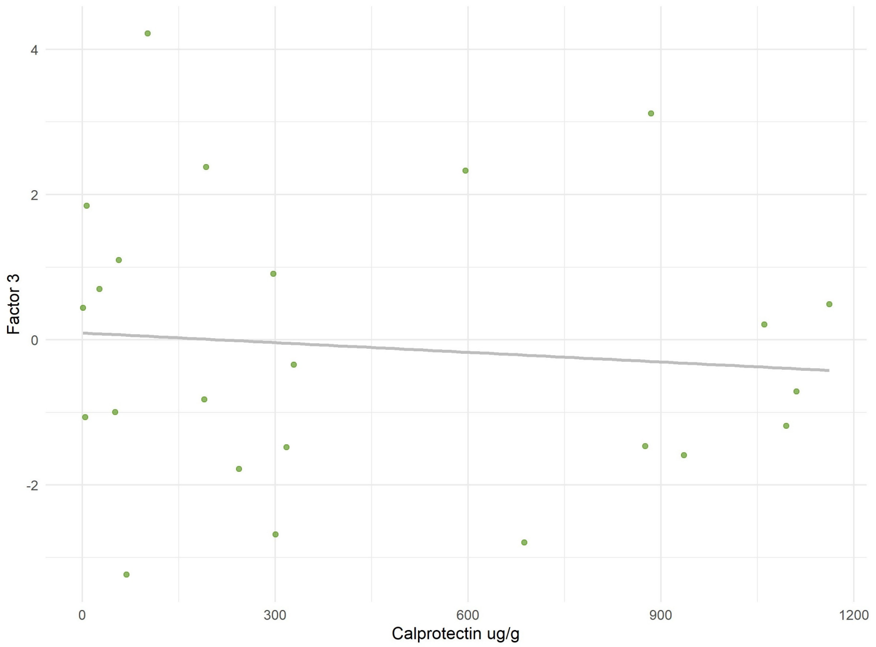

Figure S11. Factor 3-Calprotectin
